# Role of corticotropin-releasing factor on bladder function in rats with psychological stress

**DOI:** 10.1038/s41598-019-46267-9

**Published:** 2019-07-08

**Authors:** Masaya Seki, Xin-Min Zha, So Inamura, Minekatsu Taga, Yosuke Matsuta, Yoshitaka Aoki, Hideaki Ito, Osamu Yokoyama

**Affiliations:** Department of Urology, Faculty of Medical Science, University of Fukui, Fukui, Japan

**Keywords:** Bladder, Neurophysiology, Peptide hormones

## Abstract

Stress-related peptide corticotropin-releasing factor (CRF) and CRF-related peptides are distributed in the peripheral viscera such as the bladder. We investigated the contribution of psychological stress (PS) and CRF on bladder function. Male rats received sham stress (SS) or PS using a communication box method for 120 min every day for 7 days. One group of rats received the intraperitoneal CRF-R1 antagonist antalarmin for 7 days during stress exposure. Mean voided volume per micturition was significantly lower in PS rats compared to SS rats, which was antagonized by antalarmin treatment. Increases in plasma and bladder CRF, and mRNA expressions of bladder CRF, CRF-R1, and M2/3 muscarinic receptors, were found in PS rats. CRF did not influence bladder contraction in itself; however, stress increased the response of muscarinic contraction of bladder strips. These changes were antagonized by antalarmin treatment. In conclusion, PS reinforces M3 receptor-mediated contractions via CRF-R1, resulting in bladder storage dysfunction.

## Introduction

Corticotropin-releasing factor (CRF), isolated from the hypothalamus, is a main factor of the hypothalamic-pituitary-adrenocortical (HPA) axis and is known as stress-related peptide^1^. Psychological stress influences peripheral visceral motility and sensation, and possibly induces pathological states such as irritable bowel syndrome, overactive bladder, and interstitial cystitis/bladder pain syndrome (IC/BPS)^2–7^. Activation of the CRF signaling system plays a role in numerous stress-induced behavioral and visceral disorders. This process is regulated by CRF and several CRF-related peptides, the urocortins (Ucns; Ucn1, Ucn2, and Ucn3), that bind to CRF receptors^8^. CRF and Ucns exert their action via the activation of two receptors, CRF-R1 and CRF-R2. CRF has high affinity for CRF-R1 and a ten-fold lower affinity for CRF-R2^9^.

The underlying mechanism by which CRF influences bladder function has been proposed to be in the descending pathway from the nucleus of Barrington to the sacral parasympathetic nerve (SPN) of the spinal cord^2,10,11^. Intrathecal administration of CRF decreases the magnitude of Barrington’s stimulated bladder contractions^11^, meaning that spinal CRF has an inhibitory influence on the micturition reflex. A potential mechanism that can be recruited by psychological stress to produce urinary retention has been suggested to inhibit neural circuits linking the brain and bladder that are sensitive to stress^12^.

As well as the nervous systems, CRF and CRF-related peptides are distributed in the peripheral viscera such as skin, gastrointestinal tract, pancreas, adrenal glands, and bladder^6,13^; however, their relevance in stress-induced alterations of visceral function is not well understood. CRF and CRF-related peptides are ancient developmental signaling molecules that allow developing organisms to coordinate physiological responses for adaptation, development, and survival in a changing environment^14^. These peptides and receptors can activate a plethora of signal-transduction pathways such as PKA, PKC, PKB/Akt, ERK, and p38 MAPK. In fact, CRF increases colonic contractions in *in vivo* and *in vitro* studies of rats, which means that peripheral CRF-R1 signaling enhances colonic motility^7^. In the present study, we investigated the contribution of psychological stress and CRF on bladder function in rats. We hypothesized that psychological stress also affects bladder function not only via spinal CRF, but via bladder CRF.

## Results

### Corticosterone levels in plasma

In order to test whether a communication box method was effective in administering psychological stress, plasma corticosterone levels were measured as a preliminary experiment. A significant difference (p = 0.019) was seen between rats exposed to sham stress (n = 12) and rats exposed to psychological stress (n = 12; 644.5 ± 83.6 ng/ml and 540.8 ± 122.5 ng/ml, respectively). Therefore, we considered this experimental method to be useful as a stress loading method.

### Body weight, bladder weight, and micturition parameters

**Figure 1:**
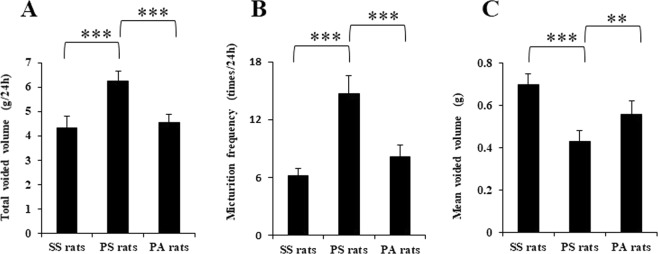
Micturition parameters in the 3 treatment groups. (**A**) total voided volumes for 24 h in sham stress (SS), psychological stress (PS), and psychological stress treated with antalarmin (PA) rats (n = 8: each). (**B**) micturition frequency for 24 h in the 3 treatment groups (n = 8: each). (**C**) mean voided volume per micturition in the 3 treatment groups (n = 8: each). Values represent means ± SEM. Double asterisks indicate statistically significant differences of p < 0.01, and triple asterisks indicate statistically significant differences of p < 0.001.

Body weights of rats exposed to sham stress (SS; n = 8), rats exposed to psychological stress (PS; n = 8), and rats exposed to psychological stress treated with the intraperitoneal CRF-R1 antagonist antalarmin (PA; n = 8) were 198.3 ± 7.6, 195.0 ± 10.9, and 195.0 ± 10.1 g after 1-week exposures to sham or psychological stress, respectively. No significant differences were found between the 3 treatment groups. Bladder weights of SS, PS, and PA rats were 78.7 ± 2.2, 78.7 ± 2.1, and 79.7 ± 2.1 mg after a 1-week exposure to sham or psychological stress, respectively. No significant differences were found between the 3 treatment groups. Voided volume and micturition frequency per day were significantly higher in PS rats compared to SS rats (both p < 0.001, Fig. 1A,B). Voided volume and micturition frequency per day were significantly lower in PA rats than in PS rats (both p < 0.001, Fig. 1A,B). Mean voided volume per micturition was significantly lower in PS rats compared to SS rats (p < 0.001, Fig. 1C). Mean voided volume per micturition was significantly higher in PA rats than in PS rats (p < 0.01, Fig. 1C).

### CRF in the plasma and bladder wall

**Figure 2:**
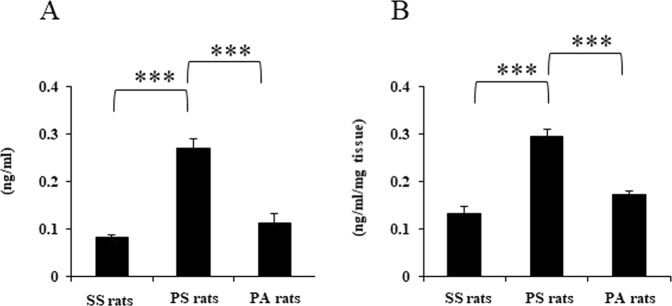
Protein amounts of CRF in the plasma (**A**) and bladder wall (**B**) of 3 treatment groups: sham stress (SS), psychological stress (PS), and psychological stress treated with antalarmin (PA) (n = 8: each). Values represent means ± SEM. Triple asterisks indicate statistically significant differences of p < 0.001.

The protein level of plasma CRF was significantly higher in PS rats compared to SS rats (p < 0.001, Fig. 2A). The protein level of plasma CRF was significantly lower in PA rats than in PS rats (p < 0.001, Fig. 2A). The protein level of bladder CRF was significantly higher in PS rats compared to SS rats (p < 0.001, Fig. 2B). The protein level of bladder CRF was significantly lower in PA rats than in PS rats (p < 0.001, Fig. 2B).

### Relative expression of mRNA in the bladder

Significant increases in mRNA expressions of CRF, CRF-R1, M_2_, and M_3_ muscarinic receptors were observed in the bladders of PS rats (p < 0.01 for all, Table 1); however, there was no change in adrenergic receptor α_1A/B/D_. Marked decreases in mRNA expressions of CRF, M_2_, and M_3_ were found in PA rats (p < 0.05 for all, Table 1).

**Table 1:** Relative expressions of mRNA of CRF, CRF receptor 1, M_2_/M_3_ muscarinic receptors, and alpha_1a/1b/1d_ adrenoceptors in the bladder.

	SS rats	PS rats	PA rats
CRF	1	4.73 ± 1.00**	2.54 ± 1.42^†^
CRF receptor 1	1	3.91 ± 1.36**	2.10 ± 0.85
M_2_ muscarinic receptor	1	5.31 ± 1.39**	3.53 ± 1.04^†^
M_3_ muscarinic receptor	1	3.30 ± 0.77**	2.08 ± 0.53^†^
Alpha_1a_ adrenoceptor	1	1.03 ± 0.13	0.94 ± 0.16
Alpha_1b_ adrenoceptor	1	1.07 ± 0.22	0.84 ± 0.26
Alpha_1d_ adrenoceptor	1	1.10 ± 0.32	0.94 ± 0.30

Values are expressed as the means ± standard error of the mean. ^**^p < 0.01 vs. SS rats, ^†^p < 0.05 vs^.^ PS rats.

### Contractile responses of bladder strips to CRF, phenylephrine, and carbachol

**Figure 3:**
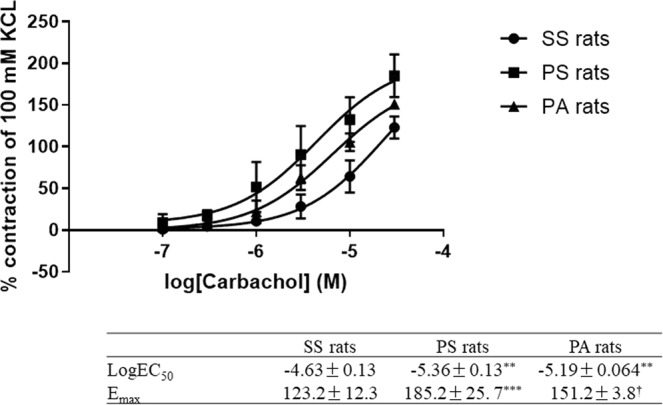
Contractile responses of bladder strips to cumulative doses of carbachol in 3 treatment groups: sham stress (SS; ●), psychological stress (PS; ■), and psychological stress treated with antalarmin (PA: ▲) (n = 8: each). ^**^p < 0.01 vs. SS rats, ^***^p < 0.001 vs. SS rats, and ^†^p < 0.05 vs. PS rats.

Exogeneous CRF did not induce the contraction of bladder strips, and the CRF-R1 antagonist antalarmin did not decrease it in the present study. The maximum carbachol contraction of the PS rat bladder was significantly higher than that of the SS rat (p < 0.001, Fig. 3), and conversely it decreased in the antalarmin treatment group (p < 0.05, Fig. 3). The EC_50_ was significantly different between SS and PS rats, and between SS and PA rats (both p < 0.01, Fig. 3), meaning that the reactivity to carbachol was elevated in the PS rats.

## Discussion

The present study shows bladder storage dysfunction in rats in response to psychological stress exposure using a communication box method. This was accompanied by increases in plasma and bladder CRF protein, and mRNA overexpression of CRF, CRF-R1, M_2_, and M_3_ muscarinic receptors of the bladder. CRF did not influence bladder contraction itself; however, psychological stress increased muscarinic contractions of the bladder strips. These contractions were partially prevented by the CRF-R1 antagonist antalarmin, which was administered during the stress exposure. Antalarmin also inhibited the increase in plasma and bladder CRF protein, and overexpression of CRF, M_2_, and M_3_ muscarinic receptor mRNA. These results support the hypothesis that CRF reinforces M_3_ receptor-mediated contractions through CRF-R1. We conclude that psychological stress influenced bladder function via the CRF signaling pathway. To the best our knowledge, this is the first study to reveal the upregulation of the local (bladder) CRF signaling pathway in response to psychological stress, which influenced muscarinic contractions of the bladder.

Upregulation of bladder CRF and CRF-R2, but not CRF-R1, has been shown in rat models of cyclophosphamide-induced cystitis^15^. In contrast, CRF-R1 upregulation has been reported in mouse bladder in response to lipopolysaccharide-induced inflammation^16^. Urothelium of normal cats and cats with feline interstitial cystitis expresses both CRF-R1 and CRF-R2^13^. Furthermore, sex and target tissue differences exist for both CRF-R1 and R2 expression^17^. In the present study, CRF-R1, but not CRF-R2, was detected in the male rat bladder in response to psychological stress. These differential findings might be due to experimental approach, species, and/or sex differences.

Many stress models have been developed to study the influences on bladder function and structure, including resident-intruder^12,18^, immobilization stress^19^, water avoidance stress^20^, and electrical foot-shock models^21^. Social stresses such as resident-intruder stress lead to urinary retention and abnormal urodynamic findings characterized by increased micturition volume per void, intermicturition interval, and residual urine^12,18^. These influences have been suggested to depend on an inhibitory role for endogenous CRF in Barrington’s nucleus regulation of the bladder. These stress paradigms have been reported to produce bladder dysfunction and target tissue abnormalities; however, similar stress has an inhibitory influence on bladder function or sometimes an excitatory influence. Depending on its intensity and duration, psychological stress has been reported to cause either bladder storage or voiding dysfunction in mice^22^.

To induce purely psychological stress without physical stress interference, we adopted a communication box method^23^. Compared to other stress methods, this method may reflect the actual stress situation in humans. In the present study, we found a repeated stress-induced excitatory influence on bladder function with overexpression of M_2_ and M_3_ muscarinic receptors. Upregulation of M_2_ and M_3_ muscarinic receptors in the bladder contributes to the increase in bladder contractility and storage dysfunction^24,25^. ATP release from cat urothelium in response to CRF occurs through a CRF-related signaling pathway, and the selective CRF-R1 antagonist antalarmin inhibits CRF-evoked ATP release^13^. Furthermore, increased levels of nerve growth factor in the male rat bladder are suspected to be related to frequent micturition due to exposure to repeated stress^26^. In the present study, ATP release from the urothelium and nerve growth factor were not monitored; however, the increase in micturition frequency and small micturition volume could be explained by the overexpression of M_2_ and M_3_ muscarinic receptors in the bladder.

Exogeneous CRF did not increase the contraction of bladder strips, and the CRF-R1 antagonist antalarmin did not decrease it in the present study, which is consistent with a previous report^27^. These results are different from rat colonic contractions, which were enhanced by exogeneous CRF in an *in vivo* and *in vitro* study^7^. Increased levels of CRF and CRF-R1 mRNA were also detected in bladders from rats exposed to psychological stress. Continuous administration of antalarmin prevented the increase of *in vitro* muscarinic contraction of the bladder and partially inhibited the overexpression of M_2_ and M_3_ muscarinic receptors. CRF itself plays a role in the activation of a plethora of signal-transduction pathways^14^. The ligands for G protein-coupled receptors are polypeptide hormones including secretin, glucagon, vasoactive intestinal peptide, CRF, and parathyroid hormone, which most often act in a paracrine or autocrine manner^14^. CRF-R1 agonists have been suggested to activate the receptors coupled to G-proteins by binding to at least two J-domain configurations^28^. M_2_ and M_3_ muscarinic receptors are Gi- and Gq-coupled receptors, respectively, and can be influenced by CRF. In fact, endogenously secreted CRF plays a role in exerting homeostatic lipogenic activity in human sebocytes^29^ and a role in conferring protection of cardiomyocytes against ischemia^30^ in an autocrine/paracrine fashion. Short-term immobilization stress leads to a decrease of the M_2_ muscarinic receptor in the heart^31^. Furthermore, in the hippocampus, the M_2_ muscarinic receptor is downregulated in vulnerable rats but upregulated in resilient rats exposed to unavoidable stress^32^. Muscarinic receptors are thought to be the first targets that respond to stress^33^. This response causes changes in organisms and leads to changes in the output system via the main routes: neurons and neuroendocrine systems.

In the present study, M_2_ and M_3_ muscarinic receptors were upregulated in the rat bladder in response to psychological stress, while increases in their gene expressions were inhibited in rats treated with the CRF-R1 antagonist antalarmin. Among CRF-R1 antagonists, antalarmin has greater affinity for CRF-R1 and has been reported to reverse anxiety-like behaviors induced by social stress^34^. These results suggest that systemic or local (bladder) CRF might indirectly overstimulate muscarinic receptor-mediated contraction of the bladder, resulting in bladder dysfunction. Therefore, CRF could be a neuromediator with brain-bladder or bladder-bladder interconnections. Antalarmin prevented the increase in CRF protein of the bladder in the present study, meaning that a CRF-R1 antagonist inhibited the CRF pathway in an autocrine fashion, followed by downregulation of M_2_ and M_3_ receptors.

Intrathecal CRF administration inhibits bladder contraction, and CRF-R antagonists increase micturition pressure, indicating an inhibitory influence of neural CRF on the micturition reflex^11^. In contrast, intraperitoneal CRF administration has been reported to induce bladder overactivity in rats^27^. Local CRF might compensate for the decrease in bladder contraction induced by central CRF and allow the micturition reflex to continue. When the balance between neural and local CRF systems collapse during psychological stress exposure, voiding or storage dysfunction may occur. Further investigations are necessary to explore the relationship between neural and local CRF interactions in the bladder, as well as the involvement of the CRF system in bladder pathophysiological phenomena such as overactive bladder and IC/PBS.

This study has inherent limitations such as the following: (1) no investigations could be carried out to evaluate the underlying mechanisms by which CRF activates M_2_ and M_3_ muscarinic receptors coupled with Gi- and Gq- proteins in the bladder. (2) Only a few mRNAs were assessed, such as CRF, CRF-R1, M_2_, and M_3_ muscarinic receptors and the adrenergic receptors α_1A,_ α_1B,_ and α_1D_. Because many other receptors are involved in bladder function, including β_3_ adrenergic receptor, it is not possible to conclude that muscarinic receptors are the exclusive stress-related receptors. To confirm whether other candidates are related to bladder function, further experiments are necessary. (3) Bladder CRF seems to increase synchronously with serum CRF. Psychological stress increases serum CRF, and apart from this, stress may directly increase bladder mitochondrial disorders with the increase in bladder CRF. Determining the mediator between serum CRF and bladder CRF is a future task.

In conclusion, psychological stress induces bladder dysfunction in rats, which was accompanied by increases in serum and bladder CRF protein, as well as mRNA overexpression of CRF-R1, M_2_, and M_3_ muscarinic receptors of the bladder. Endogenous CRF increased muscarinic contractions of the bladder strips, which were partially prevented by a CRF-R1 antagonist. These results support the hypothesis that psychological stress influences bladder function via a local CRF-muscarinic receptor signaling pathway.

## Methods

### Animals

Male Wister Kyoto (WKY; 12 weeks old) rats weighing 179–203 g (mean 186.2 ± 15.4 g, n = 48) were used for the experiments. The animals were purchased from SLC Japan (Hamamatsu, Japan). They were kept at a constant temperature of 23 °C and a humidity of 50 to 60% under a normal 12-hour light and dark schedule. Tap water and standard rat chow were freely available. All experiments were conducted according to Fukui University’s Animal Care and Use Committee guidelines, and all experimental protocols were approved by the Fukui University Ethics Commission (number 29059/2014).

### Psychological stress exposure

**Figure 4:**
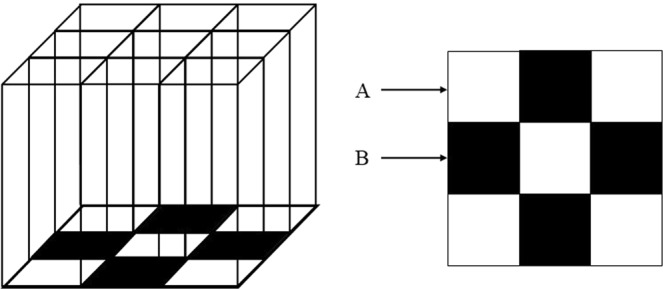
A communication box connected to an electrified shock-generator to induce psychological stress. (**A**) electrified chambers (white chambers), (**B**) stress-received chambers (black chambers).

A communication box method was developed to induce purely psychological stress without physical stress interference^23^. The communication box (CB5, Melquest Ltd., Toyama, Japan) was divided into 9 compartments (A and B) by transparent plastic sheets (Fig. 4). In compartment A, rats received electric shock (0.2 mA) for 10 s at intervals of 50 s through the floor by an electric shock generator. In compartment B, a plastic plate was placed on each of the grid floors to prevent electric shock. The rats in compartment A revealed nociceptive responses such as jumping up, defecation, and crying. On the other hand, rats in compartment B did not receive foot-shock; however, they received emotional stress from the rats in compartment A. Psychological stress was given for 120 min every day for 7 days. Animals in the sham stress group were kept in compartment B for 120 min every day for 7 days and received no psychological stress because animals in compartment A did not receive electric shocks. As a preliminary experiment, plasma corticosterone levels were measured to confirm whether this experimental method was effective for administering stress.

### Experimental groups

A preliminary experiment was performed to measure plasma corticosterone levels in rats exposed to sham stress (n = 12) and rats exposed to psychological stress (n = 12).

*In vivo* and *in vitro* studies were carried out in the following 3 treatment groups: (1) rats exposed to sham stress (SS; n = 8), (2) rats exposed to psychological stress (PS; n = 8), and (3) rats exposed to psychological stress treated intraperitoneally with the CRF-R1 antagonist antalarmin (200 μg/kg/day) for 7 days during the stress exposure (PA; n = 8).

### Measurement of micturition characteristics

Rats were kept for about 48 hours in metabolic cages, and urine was collected using a plastic box placed on an electronic balance via a urine collection funnel^35^. The cumulative weight of urine was measured every 3 minutes by an electronic balance. The values recorded during the last 24 hours were used for analysis of micturition characteristics. Based on the recorded data, 24-hour micturition frequency, mean voided volume per micturition, and 24-hour urine output were calculated.

### Measurement of CRF in the plasma and bladder wall

After measurement of micturition characteristics, the rats were killed via decapitation, and bladders were transected at the level of the ureteral orifice. We confirmed that there was no residual urine in rat bladders. Each bladder was excised and ground under liquid nitrogen. Total protein was extracted from the tissue samples by adding 1 mL of T-PER Tissue Protein Extraction Reagent (Thermo Scientific, Illinois, USA). The supernatant was used for the measurement of CRF in the bladder. Rat whole blood was collected into commercially EDTA-treated tubes. Blood cells were removed by centrifugation for 15 min at 2,000 × *g*. The resulting supernatant was used as the plasma for the measurement. CRF in the bladder and plasma were measured by an enzyme-linked immunoassay using a YK131 Mouse/RAT CRF-HS ELISA KIT (Yanaihara Research Laboratory, Shizuoka, Japan) according to the manufacturer’s instructions.

### Real-Time PCR

**Figure 5:**
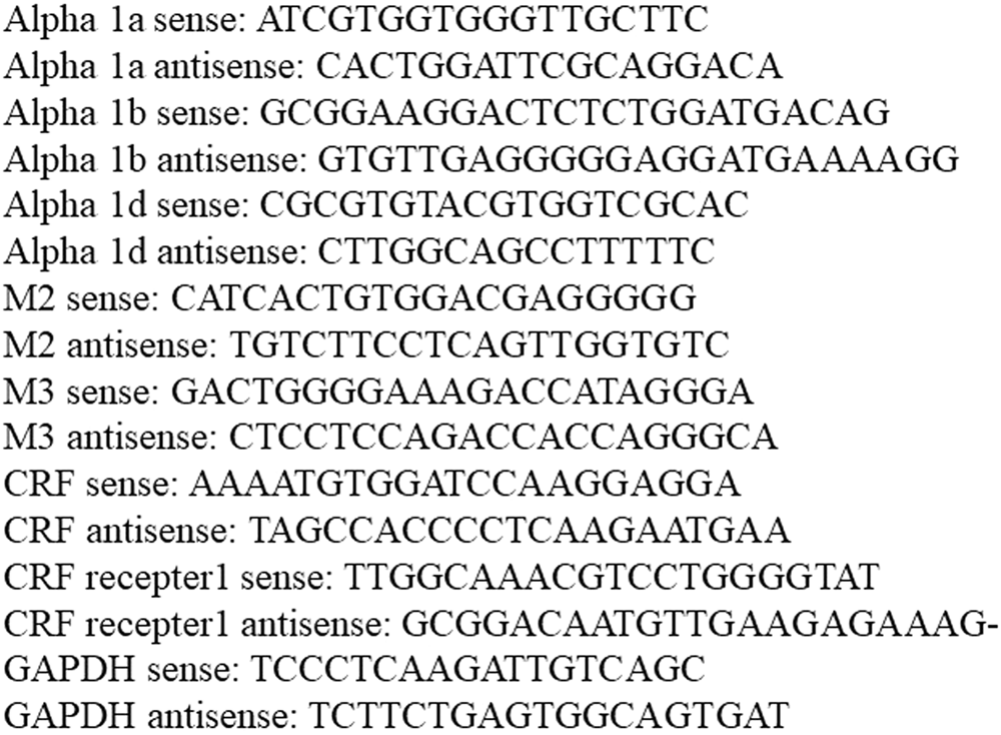
Forward primers (5′-3) and reverse primers (5′-3) used in real-time PCR.

Bladders were excised and ground into powder under liquid nitrogen. The total RNA was isolated from the tissue powder with an RNeasy Fibrous Tissue Mini Kit (QIAGEN, Tokyo, Japan) in accordance with the manufacturer’s description. RNA quality was evaluated using a Nano Drop 1000 spectrophotometer (Thermo Fisher Scientific). cDNA was synthesized from 500 μg of total RNA using a High Capacity RNA-to-cDNA Kit (Applied Biosystems, Warrington, UK) in accordance with the manufacturer’s instructions in a total reaction volume of 20 μL. The level of target mRNA expression in the bladder was analyzed using the SYBR green fluorescence relative quantification method with an ABI 7300 Real-Time PCR System (Applied Biosystems, Japan). GAPDH was used as an internal control. The PCR reaction volume consisted of 10 μL of Power SYBR Green PCR Master Mix (Applied Biosystems), 2 μL of primers, 1 μL of cDNA, and 7 μL of nuclease-free water. The forward primers (5′-3′) and reverse primers (5′-3′) used in real-time PCR are listed in Fig. 5.

### *In vitro* organ bath studies

2 × 5 mm bladder strips containing the urothelium of rats exposed to sham or psychological stress, the major axis facing to the bladder neck, were cut longitudinally from the dome of the bladder and mounted in separate 10 mL organ baths. The organ baths contained normal Krebs solution (in mM: NaCl 118.4, KCl 4.7, CaCl_2_ 1.9, NaHCO_3_ 24.9, MgSO_4_ 1.15, KH_2_PO_4_ 1.15, and glucose 11.7), bubbled with 5% CO_2_ in O_2_, and maintained at 37 °C. The strip was stretched to a resting tension of approximately 1 g and equilibrated for 45 min. Changes in tension were isometrically monitored with a force-displacement transducer connected to a PowerLab data acquisition system (ADInstruments, Nagoya, Japan). After equilibration, the maximum contraction induced by K^+^ (100 mM) was obtained by exchanging normal Krebs solution with high K^+^ Krebs solution. Changes in the isometric tension of each bladder strip for cumulative doses of carbachol (10^−7^, 3 × 10^−7^, 10^−6^, 3 × 10^−6^, 10^−5^, 3 × 10^−5^ M) were measured in the organ bath experiments. Other strips were used to measure the response to repetitive administration of CRF (0.1 μg/ml × 5).

### Statistical analysis

Results are expressed as means ± standard error of the mean (SEM). Student’s *t* test and analysis of variance (ANOVA) were used for statistical analysis where appropriate. P < 0.05 was considered statistically significant.

### Drugs and chemicals

Carbachol (carbamoylcholine chloride) and CRF were purchased from Sigma Chemical (St. Louis, MO, USA). Antalarmin (a CRF-R1 antagonist) was purchased from Santa Cruz Biotechnology (Santa Cruz, TX, USA).

### Study approval

All experiments were conducted according to Fukui University’s Animal Care and Use Committee guidelines, and all experimental protocols were approved by the Fukui University Ethics Commission (Number 29059/2014).
